# DCE-MRI Perfusion and Permeability Parameters as predictors of tumor response to CCRT in Patients with locally advanced NSCLC

**DOI:** 10.1038/srep35569

**Published:** 2016-10-20

**Authors:** Xiuli Tao, Lvhua Wang, Zhouguang Hui, Li Liu, Feng Ye, Ying Song, Yu Tang, Yu Men, Tryphon Lambrou, Zihua Su, Xiao Xu, Han Ouyang, Ning Wu

**Affiliations:** 1 Department of Diagnostic Radiology, National Cancer Center/Cancer Hospital, Chinese Academy of Medical Sciences & Peking Union Medical College, Beijing, China; 2 Department of Radiation Oncology, National Center/Cancer Hospital, Chinese Academy of Medical Sciences & Peking Union Medical College, Beijing, China; 3 University of Lincoln, Lincoln School of Computer Science, Lincoln, LN6 7TS, United Kingdom; 4 GE Healthcare, 10 Ronghua Road, Beijing, 100176, China; 5 PET-CT Center, National Center/Cancer Hospital, Chinese Academy of Medical Sciences & Peking Union Medical College, Beijing, China

## Abstract

In this prospective study, 36 patients with stage III non-small cell lung cancers (NSCLC), who underwent dynamic contrast-enhanced MRI (DCE-MRI) before concurrent chemo-radiotherapy (CCRT) were enrolled. Pharmacokinetic analysis was carried out after non-rigid motion registration. The perfusion parameters [including Blood Flow (BF), Blood Volume (BV), Mean Transit Time (MTT)] and permeability parameters [including endothelial transfer constant (K^trans^), reflux rate (K_ep_), fractional extravascular extracellular space volume (V_e_), fractional plasma volume (V_p_)] were calculated, and their relationship with tumor regression was evaluated. The value of these parameters on predicting responders were calculated by receiver operating characteristic (ROC) curve. Multivariate logistic regression analysis was conducted to find the independent variables. Tumor regression rate is negatively correlated with V_e_ and its standard variation V_e__SD and positively correlated with K^trans^ and K_ep_. Significant differences between responders and non-responders existed in K^trans^, K_ep_, V_e_, V_e__SD, MTT, BV_SD and MTT_SD (*P* < 0.05). ROC indicated that V_e_ < 0.24 gave the largest area under curve of 0.865 to predict responders. Multivariate logistic regression analysis also showed V_e_ was a significant predictor. Baseline perfusion and permeability parameters calculated from DCE-MRI were seen to be a viable tool for predicting the early treatment response after CCRT of NSCLC.

Lung cancer is the leading cause of cancer death in men and women worldwide^1^. More than 70% of non-small cell lung cancers (NSCLCs) are found as locally advanced unresectable disease or as advanced metastatic disease. Radiotherapy or concurrent chemo-radiotherapy (CCRT) has been an essential treatment modality^2^. However, even with the same clinical stage and pathological subtype, the prognosis of locally advanced NSCLC is different, which indicates tumor heterogeneity or different individual radio-sensitivity.

Blood supply to tumor is either through direct blood supply or vessel leakage. These vascular characteristics are known to influence radio-sensitivity through their effect on oxygen free radical generation, which interferes with the repair of radiation-induced DNA damage. The vascular characteristics of the tumors influence the extent of exposure to chemotherapy drugs and the level of drug activity by determining the intra-tumor pH and the ratio of quiescent cells within the tumor^3^,^4^. Various techniques can be applied to extract these hemodynamic information, such as ASL (Arterial Spin Labeling), DSC (Dynamic Susceptibility Contrast) and DCE (Dynamic Contrast-Enhanced) MRI. Unlike ASL and DSC, which are largely confined to neuroimaging, DCE-MRI can be applied to the whole body and provide both perfusion and permeability information^5^. As a non-invasive technique, DCE-MRI has been used as a predictor for tumor treatment response on many anatomies i.e. brain, breast etc^5^,^6^. DCE-MRI in lung cancer is an under-studied area, although previous DCE-MRI research was performed in lung by Naish *et al*.^7^ and CT perfusion research was performed by van Elmpt *et al*.^8^ The major difficulty for dynamic scan to be performed in lung is breathing motion. Recent research on tackling this difficulty has been laid on using medical image registration^9^, which has been successfully applied to chest CT^10^. Research has been performed and concluded that it is quite necessary to have a motion control component i.e. image registration in DCE analysis steps to ensure high parameter accuracy^11^,^12^.

We conducted a prospective study to investigate whether baseline DCE-MRI perfusion and permeability parameters can provide useful information to predict CCRT response in patients of NSCLC.

## Patients and Methods

### Patients

This single-center prospective study was approved by the Ethics Committee of Cancer Hospital of Chinese Academy of Medical Sciences, and informed consents were obtained from all patients. This study was conducted in accordance with the Declaration of Helsinki. From January 2013 to April 2015, 40 consecutive NSCLC patients staged IIIA or IIIB, who planned to receive CCRT for lung cancer in our hospital were prospectively recruited to this study. To determine the clinical stage, all of the 40 patients underwent chest CT, brain MRI, skeletal scintigraphy, and abdominal ultrasonography before therapy. The inclusion criteria were as follows: (a) biopsy-proven NSCLC, (b) the largest diameter of the pulmonary mass was 2.0 cm or larger, (c) no history of prior chemotherapy or radiotherapy or other therapy, (d) no contraindications for MR examination, and (e) agreement to participate in the study. All participants underwent DCE-MRI before CCRT within 1 week. The follow-up MR or CT scan was obtained at 1 month after the end of radiation therapy (when the total dose reached 60 Gy) to assess the tumor response to the therapy. Four patients who changed treatment regimen or terminated CCRT were excluded due to metastases or other serious disease during CCRT. Thus, the final cohort included 36 patients. Patient characteristics are listed in Table 1.

### MRI Protocol

All MR examinations were performed on a 3.0 T scanner (Discovery MR750 3.0 T, USA) by using an eight-tunnel body phased-array coil. Multi-flip angles (3°, 6°, 9 ° and 12°) were first performed in the axial plane encompassing the entire tumor volume before dynamic scanning to determine pre-contrast T1 mapping. Dynamic sequence (3-Dimensional T1W fast spoiled gradient echo with repetition time/echo time = 2.9 ms/1.3 ms, flip angle = 12°, section thickness = 4.2 mm, gap = 0 mm, section number = 24) was then performed with a 4 s tempo and continued for 168 s (42 phase). During dynamic acquisition, patient took a breath for every 12 s. After the first three dynamic scans were performed, contrast agent (Omniscan, GE Healthcare) was injected at a dose of 0.1 mmol/kg of body weight with the injecting rate of 2.0 ml/s by power injector. Then the delayed phase was performed in the axial and coronary plane, which covered the area from the thoracic inlet to the adrenal glands.

Prior to DCE-MRI, T1-and T2-weighted images were obtained in the transverse plane in each patient. Pulse-gated T1-weighted fast spin echo images (repetition time/echo time = 600–900 ms/5.8 ms, matrix = 288 × 192) and respiratory-gated T2-weighted fast spin echo images [repetition time/echo time = (6000 ms–8000 ms)/85 ms, matrix = 288 × 224] were obtained with FOV of 360 mm–400 mm, section thickness of 6 mm, and gap of 1 mm encompassing the thoracic inlet through the adrenal glands routinely.

### Image Analysis

Firstly, motion within multi-flip angle data and dynamic data were pre-processed by using a mutual-information based nonlinear registration algorithm. Quality of image registration was assessed subjectively by two reviewers (S.Y. and L.L. with 10 and 12 years of experience in MR imaging, respectively) in consensus according to a three category scoring system: A score of 3 = good, a score of 2 = moderate, and a score of 1 = poor. The rating was determined on the basis of the registration quality, possible mis-registration artifact, and architectural distortion of the tumor and vessel branches^13^. Secondly, pharmacokinetic analysis was carried out on motion corrected data, using in-house developed software, Omni-kinetics (GE Healthcare, Life Science, China). Thirdly, arterial input function (AIF) was obtained by placing a ROI on thoracic aorta on transverse plane in the peak arterial enhancement phase^14^,^15^. We used an accelerated version of Tofts model, which is the Extended Tofts Linear model^16^, to generate permeability parameters (K^trans^: endothelial transfer rate, min^−1^; K_ep_: reflux rate, min^−1^; V_e_: the fractional extracellular extravascular space (EES) volume, 0 < V_e_ < 1, dimensionless; V_p_: fractional plasma volume, 0 < V_p_ < 1) and de-convolution method with delay correction^17^ was used to generate perfusion parameters (BF: blood flow, ml/100 ml/min; BV: blood volume, ml/100 ml; MTT: mean transit time, s).

Tumor ROI was placed by two radiologists (S.Y. and L.L. with 10 and 12 years of experience in MR imaging, respectively) in consensus. They went through the scan data and selected the slice with maximum tumor area. A ROI was manually drawn around the whole tumor, where the unenhanced and enhanced images were both reviewed to determine tumor boundary and avoid the presence of large vessels, and/or necrotic area(s).

### Clinical Treatment and Tumor Response Assessment

All 36 patients underwent CCRT used stereotactic body radiation therapy with total radiation dose of 60 Gy in 30 fractions of 2 Gy each and chemotherapy with a combination of carboplatin and etoposide. The follow-up MR or CT scan was obtained at 1 month after the end of radiation therapy (when the total dose reached 60 Gy) to assess the tumor response to the therapy. Tumor size was defined as the maximum diameter measured on the largest area of whole tumor. Final tumors regression rate (%) was calculated according to the following equation: 100 × (pre-treatment size − post-treatment size)/pretreatment size.

According to the RECIST1.1 criteria^18^, tumor response after therapy was classified as follows: complete response (CR), partial response (PR), stable disease (SD) or progressive disease (PD). Patients with a CR or PR were further classified as responders; the patients with SD or PD were further classified as non-responders.

### Statistical analysis

The parameters were expressed as mean ± standard deviation (SD). The SD represents the heterogeneity of tumor parameters distribution (e.g., K^trans^_SD). Analysis was performed after ruling out zero values of K^trans^ to exclude non-perfused/necrotic regions, for which the pharmacokinetic model is not valid.

SPSS for Windows software (SPSS, version 17.0) was used for all data analysis. *Mann-Whitney U* test (MW) was used to compare parameters between responders and non-responders. The relationship between quantitative parameters and tumor regression rate after CCRT was evaluated by Spearmen’s correlation analysis. The value of parameters on predicting responders were calculated by receiver operating characteristic curve (ROC). Multivariate logistic regression analysis was conducted to find the independent variables. A *P* value less than 0.05 was considered as statistically significant. Statistical tests were based on a two-sided significance level set at 0.05.

## Results

### General

A total of 36 patients were eventually enrolled. Clinical characteristics for these patients are shown in Table 1. Mean tumor size was (4.7 ± 1.5) cm (range 2.3 cm–7.4 cm). The median interval between MRI and initial therapy was 3 days (range 1day–6 days). After CCRT, 21 patients were classified as responders and 15 patients were classified as non-responders. The mean tumor size after treatment was (2.6 ± 1.3) cm (range 0.5 cm–5.1 cm). The quality of all motion corrected images were graded as good (31/36) or moderate (5/36).

### Baseline permeability and perfusion parameters in responders and non-responders

**Figure 1:**
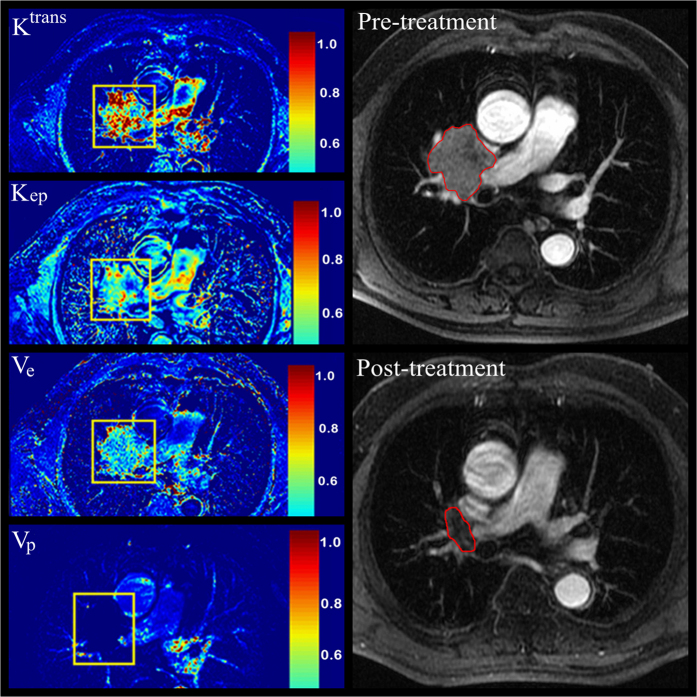
A 65-year-old man with lung squamous cell carcinoma, who partial responded to concurrent chemo-radiotherapy and classified as responders was shown (K^trans^ mean value, 0.65 min^−1^; K_ep_ mean value, 1.88 min^−1^; V_e_ mean value, 0.21; V_p_ mean value, 0.01).

The correlation between baseline permeability parameters and clinical response of NSCLC to CCRT are summarized in Table 2. Responders had higher K^trans^ and K_ep_ than non-responders, whereas responders had lower V_e_ than non-responders. Tumor regression rate after treatment was positive correlated with pre-treatment K^trans^ (*P* = 0.004) and K_ep_ (*P* = 0.041), and was negative correlated with pre-treatment V_e_ (*P* = 0.008). The baseline enhanced images and color maps of permeability parameters are shown in Fig. 1.

**Figure 2:**
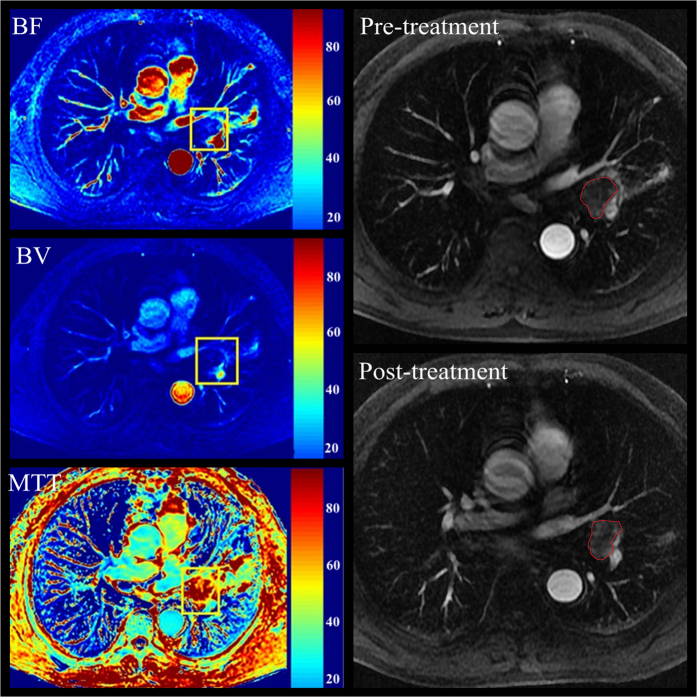
A 62-year-old man with lung adenocarcinoma, who was stable to concurrent chemo-radiotherapy and classified as non-responders was shown (BF mean value, 19.8 ml/100ml/min; BV mean value, 22.3 ml/100 ml/min; MTT mean value, 59.2 s).

The correlation between baseline perfusion parameters and clinical response of NSCLC to CCRT are summarized in Table 3. Responders had shorter MTT [(34.2 ± 8.1) s vs. (41.9 ± 8.3) s, *P* = 0.011] than non-responders. However, there was no correlation with tumor regression rate and pre-treatment perfusion parameters. Patients with lower pre-treatment BV_SD and MTT_SD tend to have a good response (*P* < 0.05). The baseline enhanced images and perfusion parameters color maps are shown in Fig. 2.

### The receiver operating characteristic curve (ROC) and multivariate logistic regression analysis of kinetic parameters

**Figure 3:**
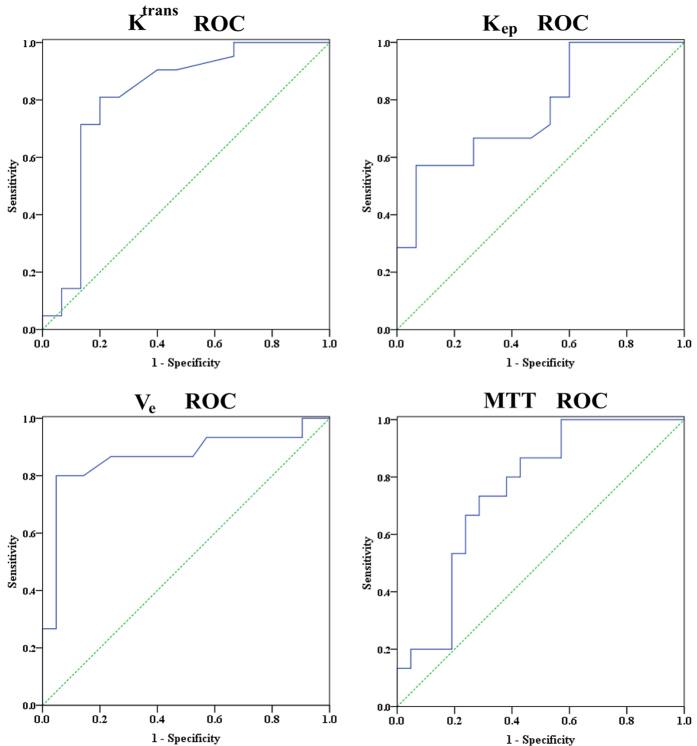
ROC curves for differentiation of responders from non-responders based on the pre-treatment K^trans^, K_ep_, V_e_ and MTT, the area under curve (AUC) was 0.808, 0.767, 0.865 and 0.752 respectively.

The permeability and perfusion parameters, which showed good prediction capabilities to distinguish between responders and non-responders, were further analyzed by using receiver operating characteristic curve as in Table 4 and Fig. 3. ROC indicated that V_e_ has the best differentiation ability. By setting threshold of V_e_ to 0.24, the specificity, sensitivity and accuracy were 85.7%, 80.0% and 83.3%, with area under curve (AUC) of 0.865 (*P* < 0.001).

The multivariate logistic regression analysis showed that V_e_ was a significant predictor for estimating the responders. Details are shown in Table 5.

## Discussion

T1W DCE-MRI has been extensively used in monitoring tumor response to antiangiogenic and vascular disrupting agents, radiotherapy and chemotherapy^6^,^19^. It appears to be a useful tool in the investigation of tumor microvascular structure and heterogeneity, which potentially improve sensitivity to subtle drug effects and provide additional understanding of tumor biology. However, this technique was not fully explored in lung cancer due to breathing motion. In our prospective study, we incorporate a medial image registration component to ensure better parameter accuracy and investigate if DCE-MRI can be a predictor of tumor response for patients with locally advanced lung NSCLC.

The pharmacokinetic parameters can be affected by the application of arterial input function (AIF)^14^,^15^. To incorporate the AIF data, several approaches have been proposed, such as reference experimentally derived AIFs, manual selection of individual AIFs, population-averaged AIFs or automatically extracted personalized AIFs^14^,^15^,^20^,^21^. Reference experimentally derived AIFs or population averaged AIFs mainly used for low temporal DCE-MRI protocol i.e. breast^22^. The application of automatically extracted personalized AIFs is confined to various factors, such as motion artifacts, the pulse of aorta, enhancing anatomical structures adjacent to the aorta and temporal resolution^14^,^15^. Therefore, we used a manual selection of individual AIFs with an ROI placed on thoracic aorta on transverse plane in the peak arterial enhancement phase. Compared with other publications in DCE-MRI on lung cancer, similar order of magnitude in parameters was obtained in our results. For example, one clinical DCE-MRI paper on lung cancer^23^, gave K^trans^ values in the range of 0.058 to 2.703 min^−1^, with a median of about 0.5 min^−1^ and close to our results. Another study^24^ achieved same order of magnitude as ours but smaller in numbers (mean K^trans^ = 0.125 min^−1^, mean K_ep_ = 1.194 min^−1^) in permeability parameters. Neither of the above two studies showed BV or MTT values.

Kelly *et al*.^25^ evaluated KRAS mutations, angiogenic biomarkers, and DCE-MRI in patients with advanced non-small-cell lung cancer receiving sorafenib. In that study, K_ep_ demonstrated a significant predictive value for overall survival (OS, *P* = 0.035) and progression free survival (PFS, *P* = 0.029). The accuracy of pharmacokinetic parameters is very sensitive to patient motion. K_ep_ is considered more robust than the other parameters in the presence of patient motion^26^. To reduce image motion in time domain, non-rigid registration was performed in our study, and subjective evaluations on image registration showed that all registered images had good or moderate image quality. From statistical results it can be seen that, many baseline perfusion and permeability parameters can predict early tumor response. Therefore, we find more statistical valuable parameters than previous Kelly *et al*.^25^ research, although our experiment is in the same organ but different end point. It is highly recommended to have a motion control component in the experiment such as image registration method.

The pre-treatment permeability parameters K^trans^ and K_ep_ were significantly higher in responders than in non-responders. These results might support the hypothesis that high K^trans^ and K_ep_ values indicate high blood supply and therefore better oxygenation and showed efficient radiation sensitivity^27^. Tumors with poor blood supply will lead to chronic hypoxia of tumor cells, which influence radio-sensitivity through their effect on oxygen free radical generation by interfering with the repair of radiation-induced DNA damage, and thereby promoting the transfer of tumor cells into subtypes with more resistance to chemotherapy and radiation regimens^28^,^29^,^30^. Tumors with a higher level of permeability are suggested to be better oxygenated, resulting in better access to the chemotherapeutic drug and better radio-sensitivity.

To date, several studies have evaluated the correlation between quantitative DCE-MRI permeability parameters and tumor response to radiotherapy in malignant tumors, and conflicting results have been reported. Zahra *et al*.^31^ reported that pre-treatment K^trans^ and K_ep_ had a significant correlation with tumor response in cervix cancer. Ahn *et al*.^32^ found that the good response group tended to show lower pretreatment K_ep_ and higher pretreatment V_e_ than the poor response group in a human colorectal cancer xenograft. However, Gu *et al*.^33^ reported in their rectal cancer study that DCE-MRI parameters at baseline were worthless to differentiate between responders and non-responders. These contrary results may due to small sample size (*n* = 5–13), different clinical staging of patients, different clinical treatment programs or other mixed factors. Our study selected locally advanced non-small cell lung cancer (stage III) with no treatment history as the object of research, and all patients conducted MR examination using the same regimen at the same time point, largely excluded the confounders above.

It is worth mentioning the value of baseline V_e_ on predicting responders, which is different to previous studies. Although Ellingsen *et al*.^34^ reported that there was no association between the values of pre-treatment V_e_ and hypoxia in cervical carcinoma xenografts, Kim *et al*.^35^ shown that the early increase of V_e_ associated with tumor regression of cervical cancer to radiotherapy. Also Cheng *et al*.^36^ had a similar result in Lewis lung carcinoma (LLC) tumor that the early increase in V_e_ and ADC correlated with tumor control. O’Connor *et al*.^37^ reported that, high median V_e_ was associated with greater CRC liver metastasis shrinkage following bevacizumab and FOLFOX-6, and argue that median V_e_ is an estimate of the extracellular extravascular space affected by factors including cell size, number, and packing density. The high median V_e_ may represents a direct estimate of the distribution space to which a contrast agent or drug can be delivered, which may indicate the potential for greater extravasations of chemotherapy and bevacizumab into the extracellular extravascular space. We assume that lower values of V_e_ indicate higher cell density and, hence, lower rates of necrosis and more viable tissue. We may explain this result using the apparent diffusion coefficient (ADC) derived from diffusion-weighted imaging (DWI), which has been shown to correlate inversely with tissue cellularity^38^. Studies of a few carcinomas have shown that cellular tumors with low baseline ADC values respond better to chemotherapy or radiation treatment than tumors with high pretreatment ADC values^39^,^40^,^41^. Although we did not find any accurate reports interpreting the relationship between V_e_ and ADC values, further studies should be performed.

From perfusion aspect, the pre-treatment values of MTT are also promising, which can predict responder on less than 37.3 s, and have negative correlation with the NSCLC regression rate to CCRT. Cho *et al*.^27^ demonstrated that the non-necrotic in the well-perfused region has a “rapid uptake and rapid washout” enhancement mode in the Dunning R3327-AT prostate tumor of rat, whereas the hypoxic regions, typically characterized by reduced vascularization, showed a delayed contrast enhancement corresponding to a delay in signal build-up and also to a delay in washout. In necrotic regions of the tumor, the time-dependent increase in the MR signal was slowest, and no washout could be observed for the duration of the MR experiment. Another study claimed the perfusion indices MTT was correlated with the micro-vascular density of malignant solitary pulmonary nodules^42^, also supports our results. BF has a *P* value 0.07, which is very close to 0.05. Hypothetically, perfusion and permeability can all be helpful in performing tumor response prediction by providing blood supply information. By using a larger data set, it is possible that BF can show statistical difference.

Heterogeneity analysis is realized by using Standard Deviation (SD). It was reported that describing heterogeneity within tumors can providing more understanding of tumor biology^19^. Aerts *et al*.^43^ demonstrated that intratumour heterogeneity was strongly prognostic, and was associated with gene-expression profiles. de Langen *et al*.^23^ indicated that patients with an increase of more than 15% in the SD of tumor K^trans^ values, which mean an increase of intra-tumor heterogeneity, predicted for treatment failure. In our study, it showed that lower values SD of V_e_, MTT and BV, mean relatively homogeneous of these parameters and predict a better prognosis. It is interesting to mention that heterogeneity analysis of K^trans^ and K_ep_ did not show any value. However, previous non-valuable parameter BV became useful by using SD analysis, which demonstrated that heterogeneity analysis can reveal previously hidden useful information.

Compared between perfusion and permeability parameters in correlations with tumor regression rate, statistical results showed that permeability (K^trans^, K_ep_, V_e_ and V_e__SD) are excellent (*P* < 0.05) predictor, whereas perfusion parameters are not related (*P* > 0.05) at all. Although, the mechanism behind the difference is not quite clear, this definitely raise the importance of using permeability as a predictor in the future.

There are several limitations in our study. Firstly, the tumor analysis on a single slice is sub-optimal. However, the tumor response assessment was performed according to the RECIST1.1 criteria, which measure the largest diameter of the largest slice. Therefore, at the current stage we just analyzed the largest slice. Secondly, the follow-up period was short, and we did not evaluate clinical end points such as overall survival rate or progression-free survival. Thus, we did not evaluate the correlation between pre-therapy DCE-MRI parameters and these endpoints. Thirdly, a comparison between perfusion and permeability parameters between tumor and healthy lung tissue would be informative for a baseline study.

In conclusion, our preliminary results suggest that baseline perfusion and permeability parameters calculated from T1W DCE-MRI were seen to be a viable tool for predicting the early response after CCRT of advanced NSCLC. Pretreatment mean value of K^trans^, K_ep_, V_e_ and MTT is potentially useful for predicting treatment response, where V_e_ has the best differentiation ability. Heterogeneity analysis on perfusion and permeability parameters showed that standard deviation of V_e_, BV and MTT also demonstrated good prediction ability. Permeability (K^trans^, K_ep_, V_e_ and V_e__SD) can be used for predict tumor regression rate.

## Additional Information

**How to cite this article**: Tao, X. *et al*. DCE-MRI Perfusion and Permeability Parameters as predictors of tumor response to CCRT in Patients with locally advanced NSCLC. *Sci. Rep.* **6**, 35569; doi: 10.1038/srep35569 (2016).

## Figures and Tables

**Table 1:** Clinical characteristics of patients (*n* = 36).

Characteristics		Number of patients, %
Age (y)	Median (range)	61 years (40–72)
Sex	Male	31 (86.1%)
	Female	5 (13.9%)
Histology	Squamous cell carcinoma	30 (83.3%)
	Adenocarcinoma	6 (16.7%)
Stage	IIIA	12 (33.3%)
	IIIB	24 (66.7%)

**Table 2:** Baseline permeability parameters and clinical response.

Permeability parameters	Tumor regression (*n* = 36)		Responders (*n* = 21)	Non-responders (*n* = 15)	Responders vs. Non-responders	
	*r* value	*P* value			*P* value	*z* value
K^trans^ (min^−1^)	0.474	**0**.**004**	0.40 ± 0.15	0.26 ± 0.15	**0**.**002**	−3.118
K_ep_ (min^−1^)	0.342	**0**.**041**	2.13 ± 0.93	1.31 ± 0.50	**0**.**004**	−2.857
V_e_	−0.435	**0**.**008**	0.20 ± 0.04	0.26 ± 0.05	**0**.**001**	−3.252
V_p_	−0.052	0.762	0.05 ± 0.03	0.05 ± 0.03	0.797	−0.258
K^trans^_SD	0.060	0.766	0.18 ± 0.07	0.22 ± 0.20	0.863	−0.201
K_ep__SD	0.176	0.381	0.89 ± 0.54	0.69 ± 0.29	0.386	−0.904
V_e__SD	−0.408	**0**.**035**	0.06 ± 0.02	0.09 ± 0.04	**0**.**005**	−2.751
V_p__SD	0.074	0.713	0.03 ± 0.02	0.03 ± 0.01	0.675	−0.439

**Table 3:** Baseline perfusion parameters and clinical response.

Perfusion parameters	Responders (*n* = 21)	Non-responders (*n* = 15)	Responders vs. Non-responders	
			*P* value	*z* value
BF (ml/100 ml/min)	46.7 ± 13.1	39.0 ± 19.2	0.070	−1.813
BV (ml/100 ml)	24.3 ± 6.5	23.4 ± 7.7	0.585	−0.546
MTT (s)	34.2 ± 8.1	41.9 ± 8.3	**0**.**011**	−2.551
BF_SD	13.5 ± 5.2	14.5 ± 5.4	0.639	−0.479
BV_SD	5.8 ± 2.8	9.6 ± 2.2	**0**.**002**	−3.007
MTT_SD	8.2 ± 4.5	14.0 ± 3.9	**0**.**001**	−3.045

**Table 4:** Values of baseline parameters on predicting responders.

Parameters	AUC	Threshold	Sensitivity (%)	Specificity (%)	Accuracy (%)
K^trans^ (min^−1^)	0.808	0.28	76.2	80.0	77.8
K_ep_ (min^−1^)	0.767	1.60	66.7	73.3	69.4
V_e_	0.865	0.24	85.7	80.0	83.3
MTT (s)	0.752	37.3	73.3	71.4	72.2

AUC, area under curve.

**Table 5:** Multivariate logistic regression model for estimating the responders.

	*P* Value	OR	95% CI for OR	
			Lower	Upper
K^trans^ (min^−1^)	0.086	0.000	0.000	6.035
K_ep_ (min^−1^)	0.667	0.449	0.012	7.289
V_e_	0.006	2.869	2.342	3.515
MTT (s)	0.977	1.002	0.850	1.183

OR, odds ratio; CI, confidence interval.
